# The Effect of Rebamipide on Ocular Surface Disorders Induced by Latanoprost and Timolol in Glaucoma Patients

**DOI:** 10.1155/2015/689076

**Published:** 2015-05-10

**Authors:** Naoto Tokuda, Yasushi Kitaoka, Akiko Matsuzawa, Junsuke Miyamoto, Shinsuke Sakae, Yasunari Munemasa, Hitoshi Takagi

**Affiliations:** Department of Ophthalmology, St. Marianna University School of Medicine, Kanagawa 216-8511, Japan

## Abstract

*Purpose*. To examine the efficacy of ophthalmic rebamipide suspensions on ocular surface disorders induced by antiglaucoma eye drops. *Patients and Methods*. Forty eyes of 40 patients receiving latanoprost (0.005%) and timolol (0.5%) were included in this randomized prospective study. The patients were randomly divided into two groups (*n* = 20): the rebamipide-treated group and control group. Changes in intraocular pressure, tear film break-up time (TBUT), and corneal epithelial barrier function were evaluated at baseline, 4 weeks, and 8 weeks after rebamipide administration. Furthermore, superficial punctate keratopathy severity was evaluated by scoring the lesion area and density. *Results*. There was no significant difference in intraocular pressure before and after rebamipide treatment. However, corneal epithelial barrier function improved significantly 4 and 8 weeks after rebamipide treatment. TBUT was partially, but significantly, increased (*P* = 0.02) 8 weeks after rebamipide treatment, whereas no significant change was observed at 4 weeks. Additionally, a significant decrease in area and density of keratopathy was observed 8 weeks after rebamipide treatment but not at 4 weeks. The control group showed no significant difference compared to baseline. *Conclusions*. Our data suggests that rebamipide treatment may reduce the occurrence of drug-induced ocular surface disorder.

## 1. Introduction

Ocular surface diseases occur in 15% of elderly patients and 48–59% of patients with glaucoma [1–3]. Ocular surface disease is hypothesized to occur in patients with glaucoma due to the side effects of antiglaucoma eye drops or glaucoma surgery. Antiglaucoma eye drops are associated with a decrease in lacrimal fluid and disturbances in corneal epithelial barrier function [4]. When drug-induced corneal disorders occur, the use of antiglaucoma eye drops is generally stopped; however, this can lead to intraocular pressure elevation. Thus, it would be preferable to improve drug-induced corneal disorders without discontinuing the use of antiglaucoma eye drops.

Ophthalmic rebamipide suspensions are approved therapeutic agents for dry eye in Japan. After systemic oral intake, rebamipide increases mucin secretion from the gastric mucus, and it has been used clinically to treat gastritis and gastric ulcers [5–7]. Because of its effects on gastric mucin secretion, it was hypothesized that rebamipide might enhance mucin production in conjunctival goblet cells. Indeed, previous studies indicate that mucin production in conjunctival goblet cells was increased after rebamipide treatment [8, 9]. Given that dry eye is caused by decreased mucin levels on the ocular surface and impaired stabilization of the aqueous layer [10], rebamipide was developed as a treatment for dry eye. Although a recent study demonstrated that rebamipide ophthalmic suspensions were effective in treating keratoconjunctivitis sicca in patients with Sjögren's syndrome [11], its effect on the ocular surface condition in glaucoma patients who are using antiglaucoma eye drops remains unclear.

The purpose of this randomized and prospective study was to examine the effect of rebamipide eye drops to prevent antiglaucoma eye drop-induced corneal disorders.

## 2. Materials and Methods

Forty eyes of 40 glaucoma patients (mean age: 62.8 ± 13.1 years) were used upon meeting the diagnostic criteria for dry eye [12]. The inclusion criteria for participating in the present study were as follows: history of treatment with 0.005% latanoprost containing benzalkonium chloride (BAK) and 0.5% timolol containing BAK (unfixed combination) for six or more months, fluorescein staining score of 2 or more points, and symptoms, such as eye discomfort. The eyes were randomized into two groups: 20 eyes of 20 patients (mean age 61.4 ± 14.2 years; 8 men, 12 women) received ophthalmic suspensions containing rebamipide, while 20 eyes of 20 patients (mean age 64.3 ± 12.1 years; 13 men, 7 women) did not receive rebamipide (control). For the rebamipide treatment group, the subjects received rebamipide eye drops four times per day every day during the observation period.

Intraocular pressure (IOP) was measured by a single examiner (NT) throughout the examination period with a Goldmann applanation tonometry at 11:00 am in a sitting position.

To evaluate corneal epithelial barrier function, a slit lamp fluorophotometer for the anterior eye was used (Kowa, FL-500, Tokyo, Japan). According to the method by Yokoi and Kinoshita [13], the background fluorescence intensity of the central cornea was measured. Using a micropipette, 0.5% fluorescein sodium solution dissolved in BSS PLUS (3 *μ*L, Alcon, Fort Worth, TX, USA) was applied to the lower conjunctival sac without contact. Eyes were washed with BSS PLUS (20 mL) 10 min after application. Fluorescein uptake was measured 30 min after application using the same protocol used in the baseline measurement. The background was subtracted, and the fluorescein uptake concentration was calculated based on a standard curve as a built-in function of the FL-500. The data were expressed as ng/mL (normal value: 28 ± 16 ng/mL). For example, over 50 ng/mL indicates corneal epithelial barrier dysfunction [14].

Slit lamp microscopy was used to measure corneal status and tear film break-up time (TBUT) [15]. To measure TBUT, fluorescein sodium was applied to the eye, and the patient blinked several times to allow for uniform distribution. The time until dry spots occurred in the cornea of the open eye was measured three times, and the mean was used. The severity of superficial punctate keratopathy was evaluated by area-density (AD) classification [16], which scores the range of the lesion (area) and the density of the spotted stain.

Data were analyzed using IBM SPSS Statistics 21 (IBM Corporation, Poughkeepsie, NY, USA). Each examination was analyzed using a paired *t*-test, and a *P* value less than 0.05 was considered statistically significant. This study was performed after the approval of the ethical committee of our hospital (ethical committee approval number: 1933), and all the patients provided written informed consent.

## 3. Results

**Figure 1:**
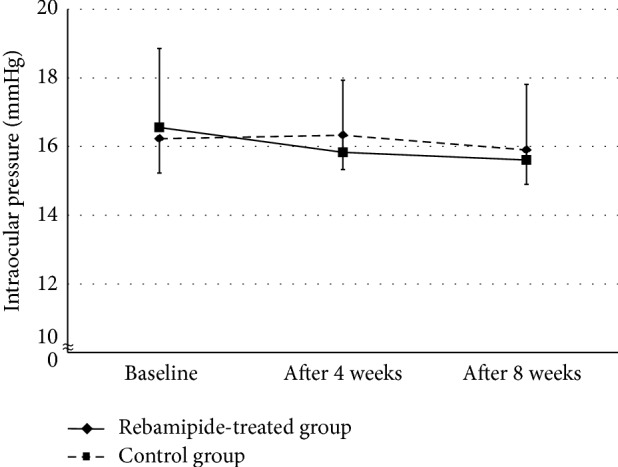
Changes in intraocular pressure in the rebamipide-treated group and control group. Both groups maintained reduced intraocular pressure without significant difference during follow-up.

We first assessed changes in intraocular pressure in patients undergoing control or rebamipide treatment (Figure 1). Rebamipide treatment had no effect on the intraocular pressure in patients receiving antiglaucoma eye drops (16.6 ± 2.3 mmHg at baseline; 15.8 ± 2.3 mmHg after 4 weeks; and 15.6 ± 1.8 mmHg after 8 weeks). Furthermore, no changes in pressure were observed in the eyes of control glaucoma patients (16.2 ± 2.3 mmHg at baseline; 16.3 ± 2.1 mmHg after 4 weeks; and 15.9 ± 2.2 mmHg after 8 weeks). Importantly, both groups maintained reduced intraocular pressure without significant difference during follow-up.

**Figure 2:**
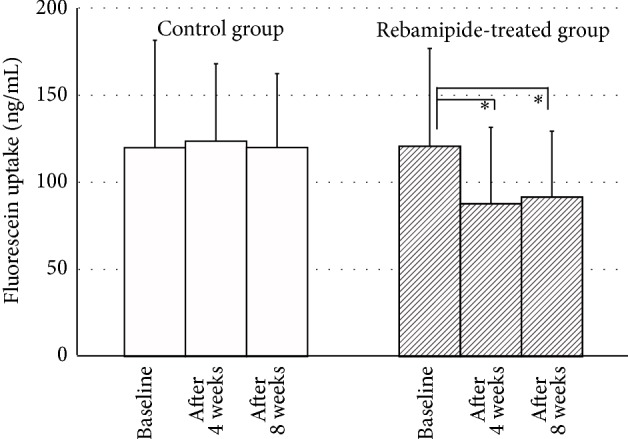
Changes in fluorescein uptake in the rebamipide-treated group and control group. Significant decreases in fluorescein uptake were observed in eyes treated with rebamipide after 4 and 8 weeks compared with baseline. ^∗^ *P* < 0.05.

We next assessed the corneal epithelial barrier function in glaucoma patients receiving control or rebamipide treatment (Figure 2). Notably, substantial increases in fluorescein uptake were observed in both groups at baseline, since both groups received 0.005% latanoprost containing BAK and 0.5% timolol containing BAK, indicating the existence of corneal epithelial barrier dysfunction induced by antiglaucoma drops. In contrast, significant decreases in fluorescein uptake were observed in eyes treated with rebamipide after 4 and 8 weeks (120.7 ± 56.1 ng/mL at baseline; 87.7 ± 43.8 ng/mL after 4 weeks [*P* = 0.012]; and 91.5 ± 37.9 ng/mL after 8 weeks [*P* = 0.017]). In contrast, no significant difference in fluorescein uptake was observed in control eyes (119.9 ± 61.7 ng/mL at baseline; 123.6 ± 44.4 ng/mL after 4 weeks [*P* = 0.671]; and 119.9 ± 42.4 ng/mL after 8 weeks [*P* = 0.995]).

**Figure 3:**
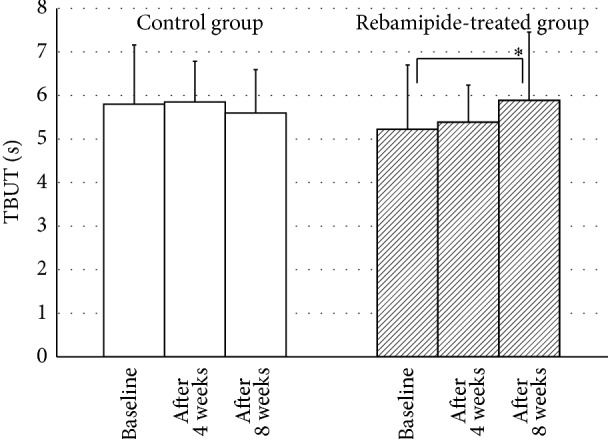
Changes in TBUT in the rebamipide-treated group and control group. Treatment with rebamipide partially but significantly increased TBUT at 8 weeks, although no significant differences were observed at 4 weeks. However, no significant difference was observed in the control group. ^∗^ *P* < 0.05.

Next, we analyzed TBUT in glaucoma patients receiving control or rebamipide treatment (Figure 3). There was no statistical difference between the control group and rebamipide-treated group at the baseline (5.8 ± 1.4 s and 5.2 ± 1.5 s, resp., *P* = 0.220). Treatment with rebamipide partially but significantly increased TBUT at 8 weeks (5.2 ± 1.5 s at baseline; 5.9 ± 1.6 s after 8 weeks, *P* = 0.02), although no significant differences were observed at 4 weeks (5.2 ± 1.5 s at baseline; 5.4 ± 0.8 s after 4 weeks, *P* = 0.331). No significant difference was observed in the control group (5.8 ± 1.4 s at baseline; 5.9 ± 0.9 s after 4 weeks, *P* = 0.771; and 5.6 ± 1.0 s after 8 weeks, *P* = 0.082).

**Figure 4:**
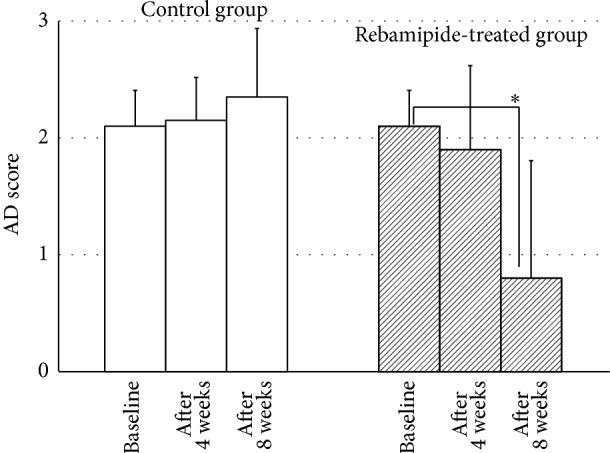
Changes in AD score in the rebamipide-treated group and control group. Treatment with rebamipide resulted in significant improvements in keratopathy at 8 weeks, but there were no changes at 4 weeks. However, no significant difference was observed in the control group. ^∗^ *P* < 0.001.

Finally, we assessed superficial punctate keratopathy in glaucoma patients by AD classification (Figure 4). Treatment with rebamipide resulted in significant improvements in keratopathy at 8 weeks, but there were no changes at 4 weeks (2.1 ± 0.7 points at baseline; 1.9 ± 0.7 points after 4 weeks, *P* = 0.162; and 0.8 ± 1.0 points after 8 weeks, *P* < 0.001). However, no significant difference was observed in the control group (2.1 ± 0.7 points at baseline; 2.2 ± 0.4 points after 4 weeks, *P* = 0.66; and 2.4 ± 0.6 points after 8 weeks, *P* = 0.06).

## 4. Discussion

Ophthalmic rebamipide suspensions are sterilized, single-use disposable therapeutics that lack preservatives to prevent secondary pollution. Thus, rebamipide is expected to have a beneficial effect on the ocular surface. Therefore, we attempted to evaluate its effect in view of several ocular surface factors in antiglaucoma eye drops-induced corneal disorder.

Fluorophotometry is a technique that can evaluate corneal epithelial barrier function. In this method, enhanced uptake of fluorescein indicates decreased corneal epithelial barrier function. Using this method, previous studies demonstrated the effects of antiglaucoma eye drops containing preservatives, such as BAK, on corneal epithelial barrier function [4, 13, 14, 17–19]. For example, a previous study showed that fluorescein uptake was significantly increased upon exposure to either timolol with BAK or timolol without BAK (baseline and postexposure values: 37.5 and 82.0 ng/mL, resp., *P* < 0.001 for preserved timolol, and 35.4 versus 57.6 ng/mL, resp., *P* < 0.001 for unpreserved timolol), and it also showed that preserved timolol exerted a greater effect (*P* = 0.028) in healthy volunteers [14]. A different study showed that although the difference in corneal fluorescein uptake was not significant, it increased from 31.3 ± 33.0 ng/mL to 72.3 ± 74.9 ng/mL in eyes treated with timolol solution with BAK (*P* = 0.073) in healthy volunteers [18]. Since timolol with BAK alone affects corneal epithelial barrier function even in the healthy subjects, it is reasonable to expect that timolol with BAK and other antiglaucoma eye drops with BAK further decrease corneal epithelial barrier function in glaucoma patients. Indeed, Ishibashi et al. [19] reported that fluorescein uptake was higher when eyes were treated with a combination of latanoprost and BAK plus *β*-blockers and BAK (118.9 ± 25.9 ng/mL) than when eyes were treated with latanoprost with BAK alone (57.1 ± 11.0 ng/mL) after 30 days. Furthermore, a report by Nakagawa et al. [20] indicated that latanoprost prepared with or without BAK can reduce corneal epithelial barrier function, implying that latanoprost itself also affects barrier function. Consistent with these findings, in the present study, we found that the group treated with timolol maleate and latanoprost showed exceedingly high fluorescein uptake at baseline in both the rebamipide-treated group and the rebamipide-untreated group (120.7 ± 56.1 ng/mL and 119.9 ± 61.7 ng/mL, resp.). These findings suggest that corneal epithelial barrier function was decreased by latanoprost with BAK and timolol maleate with BAK.

In the present study, we found that fluorescein uptake was significantly decreased in eyes treated with rebamipide after 4 or 8 weeks, as compared to baseline. Another group recently demonstrated that rebamipide increases barrier function in a human corneal epithelial cell line, as measured by transepithelial electrical resistance [21]. Therefore, it is likely that rebamipide can increase corneal barrier function* in vivo* and* in vitro*. The* in vitro* study also demonstrated the anti-inflammatory effects of rebamipide because rebamipide inhibited increases in interleukin- (IL-) 6 and IL-8 induced by tumor necrosis factor (TNF) [21]. These data suggest that antiglaucoma eye drop-induced corneal disorder is associated with inflammation because eyes treated with latanoprost with BAK and timolol with BAK had a higher mean number of inflammatory cells than eyes treated with artificial tears in the epithelium and superficial stroma in rabbits [22].

Furthermore, TBUT is widely used as a parameter to noninvasively evaluate the stability of the tear layer [15]. In a report by Kinoshita et al. [23], ophthalmic rebamipide suspensions were administered in patients with dry eye, resulting in a significant increase in TBUT versus the placebo group. Antiglaucoma eye drop-related corneal disorders are caused by decreased TBUT [2, 24, 25] and a reduction in goblet cell density [26, 27]. Timolol maleate is reported to decrease lacrimal fluid secretion through its local anesthetic effect [28] and through its toxic effect on the keratoconjunctival epithelium [29]. Both *β*-adrenergic receptor blockers and prostaglandin analogs as well as reductions in corneal sensitivity can decrease lacrimal fluid. The impact of antiglaucoma eye drops on the cornea is great because these drugs are administered without dilution. Repeated exposure of the cornea to antiglaucoma eye drops can enhance inflammatory cytokines, such as TNF, IL-1*β*, IL-6, and IL-8, thereby impairing corneal epithelial barrier function [30–33], leading to drug-induced ocular surface disorders. In the present study, we found that rebamipide had no effect on intraocular pressure but it decreased fluorescein uptake and AD score as well as enhancing TBUT. These results suggest that ophthalmic rebamipide suspensions may improve ocular surface disease as well as corneal epithelial barrier function while allowing for the maintenance of intraocular pressure with antiglaucoma eye drops. The limitations of the current study include a small sample size and a relatively short follow-up duration. Because antiglaucoma eye drops need to be continued for long periods, the effect of rebamipide should be evaluated for longer times.

In conclusion, these findings suggest that ophthalmic rebamipide suspensions may improve dry eye and repair drug-induced keratopathy when antiglaucoma eye drop-induced ocular surface disorder occurs.
